# Tinnitus functional index: validation of the German version for Switzerland

**DOI:** 10.1186/s12955-017-0669-x

**Published:** 2017-05-05

**Authors:** Nicole Peter, Tobias Kleinjung, Raphael Jeker, Martin Meyer, Richard Klaghofer, Steffi Weidt

**Affiliations:** 1 Department of Otorhinolaryngology, University Hospital of Zurich, Frauenklinikstrasse 24, CH-8091 Zurich, Switzerland; 2 Department of Psychology, University of Zurich, Andreasstrasse 15, CH-8050 Zurich, Switzerland; 3 Department of Psychiatry and Psychotherapy, University Hospital of Zurich, Haldenbachstrasse 18, CH-8091 Zurich, Switzerland

**Keywords:** Tinnitus, Tinnitus functional index (TFI), Tinnitus handicap inventory (THI), Validation, Beck depression index (BDI), Beck anxiety index (BAI), Subjective tinnitus loudness

## Abstract

**Background:**

Different standardized questionnaires are used to assess tinnitus severity, making comparisons across studies difficult. These questionnaires are also used to measure treatment-related changes in tinnitus although they were not designed for this purpose. To solve these problems, a new questionnaire - the Tinnitus Functional Index (TFI) - has been established. The TFI is highly responsive to treatment-related change and promises to be the new gold standard in tinnitus evaluation. The aim of the current study was to validate a German version of the TFI for a German-speaking population in Switzerland.

**Methods:**

At the ENT department of the University Hospital Zurich, 264 subjects completed an online survey including the German version for Switzerland of TFI, Tinnitus Handicap Inventory (THI), Beck Depression Inventory (BDI), Beck Anxiety Inventory (BAI) and sociodemographic variables. Internal consistency of the TFI was calculated with Cronbach’s alpha coefficient. Pearson correlation coefficients were used for the test-retest reliability of the TFI and to investigate convergent and discriminant validity between the THI and the BDI and BAI, respectively. Factor analysis was assessed using a principal component analysis with oblique rotation. The different factors extracted were then compared with the original questionnaire.

**Results:**

The German version of the TFI for Switzerland showed an excellent internal consistency (Cronbach’s alpha of 0.97) and an excellent test-retest reliability of 0.91. The convergent validity with THI was high (*r* = 0.86). The discriminant validity with BAI and BDI showed moderate results (BAI: *r* = 0.60 and BDI: *r* = 0.65). In the factor analysis only five factors with one main factor could be extracted instead of eight factors as described in the original version. Nevertheless, relations to the original eight subscales could be demonstrated.

**Conclusion:**

The German version of the TFI for Switzerland is a suitable instrument for measuring the impact of tinnitus. The reliability and validity of this version are comparable with the original version of the TFI. Although this study showed only five factors in the factor analysis, relations to the original eight subscales were identified. Therefore, the German version of the TFI for Switzerland can deliver relevant information regarding the different tinnitus domains.

**Trial registration:**

Clinical trial registration number on clinicaltrial.gov: NCT01837368.

## Background

Tinnitus is an auditory perception of sound in the absence of a corresponding external acoustic stimulus. Chronic tinnitus is a frequently occurring condition with reported prevalence rates ranging from 2.4 to 20% [1]. Although most people with tinnitus either cope with or habituate to the acoustic stimulus and thus report it having no impact on their quality of life, some people suffer from more severe tinnitus and view it as a debilitating condition that affects their quality of life and causes them to seek medical evaluation [2, 3].

The lack of objective means for measuring tinnitus necessitates the use of self-report questionnaires for its evaluation [4]. To this end, several psychometric questionnaires have been developed to assess the different aspects of daily life that are affected by tinnitus, such as such as concentration, sleep, emotional distress, tinnitus annoyance, and the quality of life in general. These questionnaires enable the evaluation of tinnitus severity: for example, the validated Tinnitus Handicap Inventory (THI) categorizes the total score into five levels of tinnitus severity (slight, mild, moderate, severe and catastrophic tinnitus) [5]. A problem, however, is that these questionnaires use different scaling, wording of items, and formatting, and have not been prospectively evaluated for assessing responsiveness, which makes it difficult to compare the outcomes of different trials and to determine the effectiveness of the investigated interventions [6]. To overcome these shortcomings, the Tinnitus Research Consortium (TRC) supported the development and evaluation of a new outcome measure for tinnitus [7], with the aim of improving tinnitus research [7]. Consequently, Meikle et al. (2012) developed and validated the new Tinnitus Functional Index (TFI) at Oregon Health and Science University (OHSU) [7, 8].

The TFI was developed to provide a scaling of tinnitus severity, an identification of tinnitus domains with impact on the tinnitus severity, and a responsive measurement of change in tinnitus severity [8, 9]. By using exploratory factor analysis, the tinnitus domains were defined into eight different subscales (factors): intrusiveness, sense of control, cognition, sleep, auditory, relaxation, quality of life, and emotional distress [8, 9]. Since 2012, the TFI has been translated into different languages and these translated versions have been validated in several countries (e.g., Poland, Sweden, the United Kingdom, and the Dutch speaking population of Belgium) [2, 9–11]. However, to our knowledge there is no validated German version for Switzerland of the TFI. Such a version could be used as a standard instrument in both clinical and research settings due to the TFI’s responsiveness to treatment-related changes, its comprehensive coverage of the domains of tinnitus impact, and its other psychometric properties [8, 12, 13].

The original version of the TFI had a Cronbach’s alpha of 0.97 and a test-retest reliability of 0.78 [8, 10, 12, 13]. Furthermore, the convergent validity with the THI (*r* = 0.86) and the Visual Analogue Scale (VAS) (*r* = 0.75) showed a high correlation [8, 13], and the discriminant validity yielded good results with the Beck Depression Inventory (BDI) (*r* = 0.56) [8, 13]. Moreover, the TFI was highly responsive to treatment-related change with a larger effect size than that of the THI and VAS [8].

The aim of this prospective study was to validate the German version for Switzerland of the TFI. To this end, the internal consistency and the factor structure of the translated TFI, as well as the convergent and divergent validity between the THI, the BDI and Beck Anxiety Inventory (BAI), respectively, were analyzed.

## Methods

### Participants

This study was a prospective, non-interventional, observational trial in patients suffering from tinnitus. The study was approved by the ethical committee of the Canton of Zurich and was registered on clinicaltrial.gov (NCT01837368). All participants gave their electronic consent by ticking a consent box before starting to answer the questionnaires online.

From February 2014 until April 2016, 434 patients (176 female (41.0%)) with a primary complaint of tinnitus were asked in a personalized letter to participate in the study. These patients were transferred to the department of Otorhinolaryngology at the University Hospital Zurich for further investigation and possible treatment options for their tinnitus. Four weeks before their planned consultation, these patients were then sent a letter requesting them to complete an online survey consisting of the TFI [8], THI [5], BDI [14], BAI [15], self-perceived tinnitus loudness, and their annoyance about the tinnitus. Of the 264 subjects who completed the first online survey, 128 patients (every second patient) were randomly selected two to three weeks before their consultation to complete a second survey that included only the TFI for the purpose of retesting.

Patients were included in the study only if they reported having tinnitus for a minimum of one month, were fluent in the German language, at least 18 years old, and had sufficient computer skills to participate in an online survey.

Out of the initial 434 patients, 264 (104 female (39.4%)) completed the first survey. Fifteen patients were not included due to an insufficient knowledge of the German language and 155 patients declined to participate in the study. Out of the 264 subjects who completed the first survey, 128 were randomly selected and asked to answer the second survey. Eighty-six subjects completed the second survey within the accepted period of a minimum of 7 and a maximum of 21 days between the two surveys, and could thus be included in the test-retest reliability analysis. In 13 subjects the period between answered questionnaires was too short and in 15 subjects, too long. Fourteen patients did not answer the second survey.

### Assessment

The TFI [8] is a newly-designed questionnaire for tinnitus assessment and treatment outcome measurement. The TFI consists of 25 items with a response option on an 11-point Likert scale from 0–10, with descriptors at both ends of the scale. Questions 1 and 3 are exceptions because they are expressed in percentages ranging from zero to 100%. Before performing any calculation, these answers must be transformed onto a 0–10 scale. The overall TFI score is calculated by multiplying the mean of all answered questions by 10. A minimum of 19 questions have to be answered to calculate a valid overall TFI score. As a result, the overall TFI score ranges from zero to 100, independent of the number of answered questions. Based on data collected during the development of the TFI, the overall TFI score can be categorized into five levels of tinnitus severity [12]: not a problem (0 to 17), small problem (18 to 31), moderate problem (32 to 53), big problem (54 to 72), very big problem (73 to 100). Furthermore, the items can be grouped into 8 subscales: intrusiveness (Items (I): 1–3), reduced sense of control (I: 4–6), cognitive interference (I: 7–9), sleep disturbance (I: 10–12), auditory difficulties attributed to tinnitus (I: 13–15), interference with relaxation (I: 16–18), reduced quality of life (I: 19–22), and emotional distress (I: 23–25). The calculation of a subscale score employs the same method as is equal to the calculation for the overall TFI score (i.e., the mean of answered questions in a subscale multiplied by 10) and ranges from zero to 100.

The translation of the original English version into a German version of TFI for Switzerland was initiated by Auris Medical, a Swiss biopharmaceutical company, in cooperation with the Oregon Health & Science University (“OHSU”) using a translation-back translation procedure according to the “Principles of Good Practice for the Translation and Cultural Adaptation Process for Patient-Reported Outcomes (PRO) Measures” [13, 16]. The German version of TFI for Switzerland has been transferred into an online version. To avoid several sequence effects in the test construction [17], the items were randomized using random.org (item order: 22, 14, 24, 23, 8, 18, 16, 4, 3, 6, 25, 20, 13, 2, 21, 12, 19, 10, 7, 17, 11, 1, 15, 5, 9).

To determine convergent validity for the TFI the THI [5], and as a discriminant validity the BDI [14] and the BAI [15], respectively, were assessed.

The questionnaire used to assess the convergent validity, the THI [5], is an earlier instrument for tinnitus assessment. In our study we used the validated German version with 25 questions [18]. Each question has three response options: yes (4 points), sometimes (2 points), and no (0 points). Consequently, the THI total score ranges from zero to 100.

The BDI [14] and the BAI [15] were used to assess depressive and anxiety symptoms, respectively. Each questionnaire consists of 21 items with four response options (0 to 3) and has a total score ranging from zero to 63.

Furthermore, the patients were asked to rate their self-perceived tinnitus loudness and their annoyance about the tinnitus on an 11-point Likert scale from 0–10.

### Statistical analysis

To measure the reliability of the German version of the TFI for Switzerland, internal consistency was calculated with Cronbach’s alpha coefficient. Pearson correlations were performed for all correlations and the test-retest reliability of the TFI. The convergent validity of the TFI compared with THI, and the discriminant validity of the TFI with BDI and BAI was analyzed using Pearson correlation coefficients.

For the factor analysis, Bartlett’s test of Sphericity and the Kaiser-Meyer-Olkin (KMO) test were calculated to evaluate the appropriateness of a factor analysis [19]. A principal component analysis with oblique rotation was applied to extract different factors. To determine the number of factors, two different criteria were used. First, the scree test consisting of a scree plot with eigenvalues on the Y-axis and component numbers on the X-axis was applied. In this plot, all data points that are above the point of inflexion reflect the number of factors. To determine the point of inflexion, a horizontal line and a vertical line starting from each end of the curve are drawn [20]. Second, the Jolliffe’s criterion has a suggested cut-off for eigenvalues greater than 0.7 [21]. The significance level was set at *p* ≤ 0.05 (two sided), unless otherwise specified. SPSS (Statistical Packages for Social Sciences, version 23.0, SPSS Inc., Chicago, IL, USA) was used for all statistical analyses.

## Results

A total of 264 subjects were included in this study. The mean age of participants was 48.6 years (±13.6 SD, range 18–81 years), 104 (39.4%) were female, and the mean duration of tinnitus was 72.5 months (±101.3 SD, range 1–540 months). Further demographic and clinical characteristics are presented in Table 1.

**Table 1:** Demographic and clinical characteristics of 264 patients with tinnitus

	Mean	SD
Age, years	48.6	13.6
TFI total score (0–100)	40.65	23.20
THI total score (0–100)	43.79	23.99
BDI total score (0–63)	8.95	7.34
BAI total score (0–63)	10.53	9.38
Duration of tinnitus, month	72.5	101.3
	Number of Patients	%
Gender, female/male	104/160	39.4/60.6
Partnership, yes/no	177/87	67.0/33.0
TFI Tinnitus severity categories		
Not a problem (0 to 17)	47	17.8
Small problem (18 to 31)	60	22.7
Moderate problem (32 to 53)	78	29.5
Big problem (54 to 72)	50	18.9
Very big problem (73 to 100)	29	11.0

*TFI* Tinnitus functional index, *THI* Tinnitus handicap inventory, *BDI* Beck depression inventory, *BAI* Beck anxiety inventory, Partnership: married and unmarried relationship

**Fig. 1:**
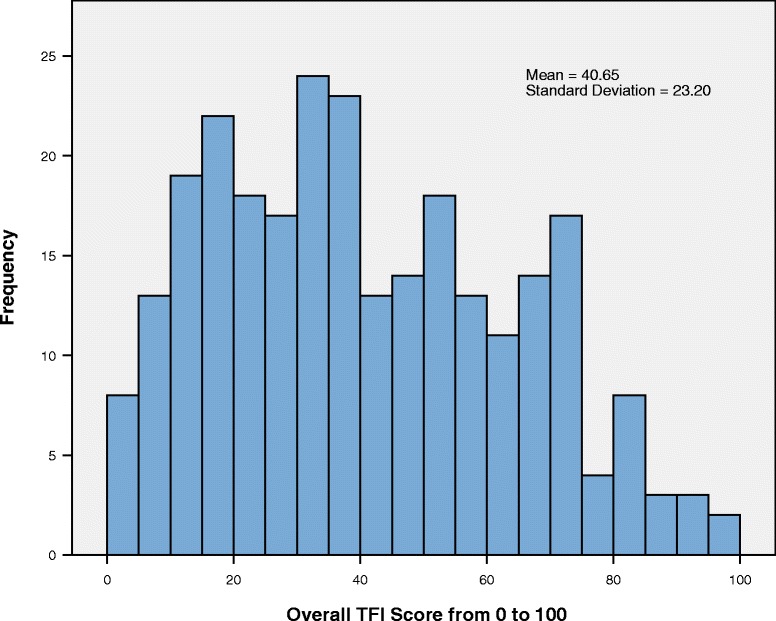
Distribution, mean and SD of the overall TFI score. The frequency of a specific overall TFI score diagramed in 5 point steps

The distribution of the overall TFI scores is displayed in Fig. 1. Cronbach’s alpha of the TFI was 0.97 and the test-retest reliability of the TFI was excellent with a reliability of 0.91 (both *p* < 0.001). The convergent validity of TFI compared with THI total score showed a high correlation of 0.86 (*p* < 0.001) and the discriminant validity of TFI when compared with BAI was 0.60, and 0.65 with BDI (both *p* < 0.001). The correlation of the overall TFI score with tinnitus annoyance was 0.71, and 0.51 with self-perceived tinnitus loudness (both *p* < 0.001).

**Fig. 2:**
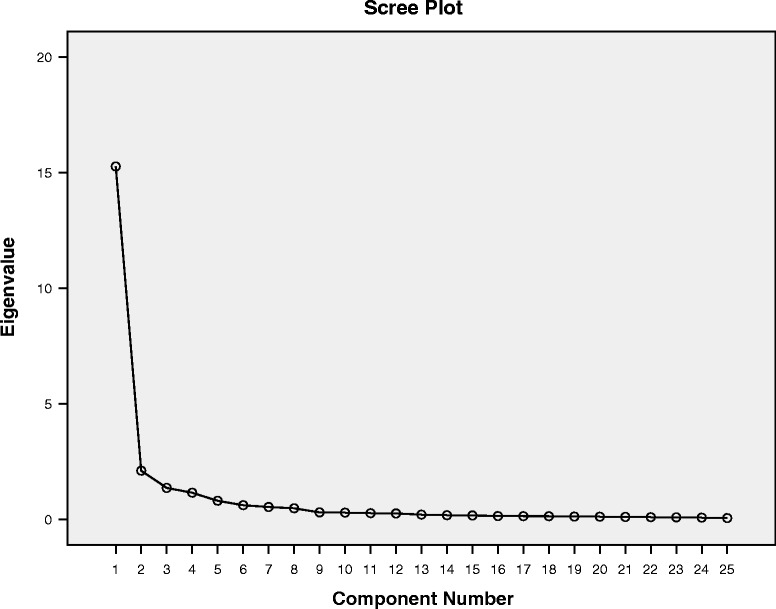
Scree Plot of principal component analysis with oblique rotation. The scree plot showed a very sharp decline after the first factor. A virtually drawn horizontal and vertical line starting from each end of the curve indicated only one factor point above the point of inflexion

The Bartlett’s test of Sphericity was significant (<0.0001), indicating that the items of the TFI were correlated. A high KMO ratio of 0.96 for the TFI implied that a factor analysis should present distinct and reliable factors. A principal component analysis with oblique rotation was applied and the resulting communalities ranged from 0.50 to 0.95. The number of components of the TFI was defined based on the eigenvalues. The scree plot showed a very sharp decline after the first factor (Fig. 2), and virtually drawn horizontal and vertical lines starting from each end of the curve indicated that only one factor point was above the point of inflexion. However, by applying Jolliffe’s criterion, a five-factor structure could be retained (Table 2) and these five factors explained 82.70% of the total variance. With reference to the original TFI’s eight factors, a principal component analysis with oblique rotation and eight fixed factors was performed (Table 3). Although the eigenvalues of the last factors were smaller than 0.7, there were correlations between the items and their factors. Only Factor 8, standing for sense of control, was represented by only one item, number 4. Items 5 and 6 were also expected to correlate with this factor but did not. Rather, Item 6 correlated strongly with Factor 1 (intrusiveness), and Item 5 did not correlate strongly with any factor. In addition, Item 22 correlated more strongly with Factor 4 (cognitive interference) than the expected Factor 7 (reduced quality of life).

**Table 2:** Principal component analysis with oblique rotation

Item	Factor				
	1 Cognitive, Quality of Life, Emotional	2 Intrusive, Sense of Control	3 Auditory	4 Sleep	5 Relaxation
8	**.739**	*.151*	*.349*	*.153*	*.272*
20	**.733**	*.383*	*.041*	*.271*	*.232*
21	**.719**	*.285*	.405	*.202*	*.028*
19	**.704**	*.284*	.459	*.190*	*.037*
25	**.693**	.471	*.135*	*.255*	*.258*
22	**.676**	*.233*	*.370*	*.206*	*.216*
7	**.648**	*.170*	*.398*	*.170*	.407
23	**.647**	.472	*.151*	*.185*	*.287*
9	**.603**	.410	*.299*	*.192*	*.315*
24	**.589**	.552	*.163*	*.201*	*.283*
1	*.251*	**.813**	*.193*	*.182*	*.153*
3	*.296*	**.774**	*.259*	*.179*	*.250*
6	*.303*	**.759**	*.134*	*.169*	*.251*
2	*.258*	**.716**	*.312*	*.181*	*.245*
5	.497	**.607**	*.200*	*.304*	*.262*
4	*.184*	**.596**	*.202*	.240	.107
13	*.225*	*.193*	**.880**	*.061*	*.138*
14	*.283*	*.211*	**.864**	*.134*	*.140*
15	*.339*	*.334*	**.807**	*.173*	*.010*
11	*.242*	*.232*	*.142*	**.885**	*.195*
10	*.198*	*.261*	*.101*	**.869**	*.247*
12	*.252*	*.231*	*.155*	**.865**	*.203*
18	*.238*	*.342*	*.121*	*.294*	**.791**
16	*.342*	*.380*	*.137*	*.310*	**.715**
17	.406	*.346*	*.113*	*.346*	**.681**
Initial Eigenvalue	15.26	2.10	1.36	1.15	.80
% of variance	61.06	8.41	5.44	4.60	3.20
Cumulative %	61.06	69.46	74.90	79.50	82.70

Appling the Jolliffe's criterion (eigenvalues >0.7) five factors could be extracted. The correlations between each item and the five factors are shown. The eigenvalues of each factor and its corresponding variance are also presented. The values less than 0.4 are italic

**Table 3:** Principal component analysis with oblique rotation with fixed eight factors

Item	Factor							
	1 Intrusive	2 Auditory	3 Sleep	4 Cognitive	5 Relaxation	6 Emotional	7 Quality of Life	8 Sense of Control
1	**.845**	*.193*	*.207*	*.114*	*.180*	*.138*	*.213*	*.045*
3	**.767**	*.251*	*.198*	*.203*	*.263*	*.164*	*.175*	*.153*
6	**.742** ^**a**^	*.111*	*.191*	*.271*	*.236*	*.181*	*.110*	**.224** ^**a**^
2	**.679**	*.326*	*.204*	*.157*	*.245*	*.259*	*.090*	*.126*
13	*.149*	**.891**	*.062*	*.177*	*.153*	*.069*	*.122*	*.086*
14	*.167*	**.872**	*.146*	*.261*	*.121*	*.158*	*.065*	*.079*
15	*.292*	**.822**	*.177*	*.198*	*.040*	*.136*	*.246*	*.088*
11	*.187*	*.143*	**.888**	*.145*	*.212*	*.119*	*.157*	*.085*
10	*.224*	*.097*	**.874**	*.138*	*.253*	*.110*	*.101*	*.086*
12	*.181*	*.155*	**.872**	*.179*	*.202*	*.159*	*.109*	*.093*
8	*.170*	*.293*	*.174*	**.754**	*.199*	*.226*	*.247*	*.106*
7	*.220*	*.327*	*.189*	**.730**	*.340*	*.095*	*.215*	*.095*
22	*.254*	*.337*	*.237*	**.657** ^**a**^	*.140*	*.303*	**.193** ^**a**^	*.030*
9	.414	*.279*	*.213*	**.514**	*.288*	*.265*	*.256*	*.067*
18	*.280*	*.143*	.296	*.178*	**.799**	*.225*	*.029*	*.060*
16	*.316*	*.151*	.303	*.220*	**.754**	*.174*	*.192*	*.118*
17	*.292*	*.118*	.339	*.281*	**.718**	*.169*	*.235*	*.116*
23	*.323*	*.214*	*.203*	*.364*	*.270*	**.679**	*.194*	*.135*
24	.400	*.231*	*.215*	*.276*	*.292*	**.635**	*.224*	*.148*
25	*.344*	*.194*	*.253*	*.298*	*.318*	**.515**	.449	*.122*
21	*.256*	.413	*.188*	*.368*	*.123*	*.160*	**.663**	*.075*
20	*.268*	*.088*	*.251*	*.285*	*.338*	*.381*	**.620**	*.137*
19	*.260*	.466	*.181*	*.383*	*.116*	*.173*	**.612**	*.059*
4	*.307*	*.193*	*.199*	*.119*	*.158*	*.163*	*.109*	**.862**
5	.485	*.254*	*.314*	*.207*	*.292*	.489	*.253*	**.151** ^**a**^
Initial Eigenvalue	15.26	2.10	1.36	1.15	.80	.61	.53	.48
% of variance	61.06	8.41	5.44	4.60	3.20	2.44	2.13	1.90
Cumulative %	61.06	69.46	74.90	79.50	82.70	85.14	87.27	89.18

The correlations between each item and the fixed eight factors are shown. The eigenvalues of each factor and its corresponding variance are also presented. Although the eigenvalues of the last factors were smaller than 0.7, there are correlations between the items and their factors. Exceptions are the following three items (marked with^a^ and as well in boldface): TFI_6, TFI_22, TFI_5. The values less than 0.4 are italic

## Discussion

The German version of the TFI for Switzerland showed an excellent internal consistency (Cronbach’s alpha of 0.97) similar to the English version (Cronbach’s alpha of 0.97), and an excellent test-retest reliability of 0.91, which exceeds that of the original version (*r* = 0.78) [8]. The convergent validity with THI was high (*r* = 0.86) and comparable to the English version (*r* = 0.75) [8]. Furthermore, the scores for tinnitus annoyance on the 11-point Likert scale correlated strongly with the TFI (*r* = 0.71). However, the correlation between self-perceived tinnitus loudness and the TFI was only 0.51. Rabau et al. [10] demonstrated correlations of a similar strength between VAS mean loudness and the TFI of 0.66, and VAS maximum loudness with the TFI of 0.59. Fackrell et al. [9] found an even weaker correlation between VAS loudness and TFI of 0.46. Similarly, other studies have also found that the correlation of subjective tinnitus loudness with THI was <0.5 [22, 23]. Nevertheless, Wrzosek et al. [11] showed a strong correlation between VAS loudness and TFI of 0.76, but also a weaker correlation with the THI of 0.61. In the original version, Meikle et al. [8] demonstrated a strong correlation of 0.75 between VAS tinnitus severity and the TFI. A possible explanation for this discrepancy could be that the two scales of tinnitus loudness and tinnitus severity measure different aspects of tinnitus.

The discriminant validity with BAI showed a moderate result of 0.60. In the original version, the discriminant validity was calculated using the BDI (*r* = 0.56) [8], and this result was slightly higher in our population (*r* = 0.65).

To follow, a factor analysis was performed to extract the different factors of the TFI. The scree plot of the German version indicated only one dominant factor with a high eigenvalue of 15.26 and all following eigenvalues ≤ 2.1. Nevertheless, using the Jolliffe’s criterion (eigenvalues >0.7), five factors could be extracted. Other studies have also failed to reproduce the original eight factor structure [2, 9–11]. For example, in the validation of the Dutch TFI [10] 7 factors were found; for the Polish TFI [11], 5 factors were identified when using a criterion for eigenvalues >1.0 (had they applied the Jolliffe’s criterion there would have been 6 factors); and in the Swedish TFI [2] validation, 6 factors were extracted. The discrepancy between our study and the original version [8] could be due to the randomization of the items and the consequential disruption of the initial order sequence with the corresponding heading used in the original paper version. To verify this hypothesis another German-speaking population should be tested with a non-randomized version.

Of our extracted five factors, Factor 1 included three subscales of the original TFI consisting of cognitive interference, reduced quality of life and emotional distress. This finding is not unexplainable because quality of life has already been found to be associated with cognitive functions and emotional distress [24]. This interpretation is further supported by findings from the validation of the Dutch TFI, in which the subscales cognitive interference and reduced quality of life were summarized in one factor [10].

To verify the eight subscales of the original TFI [8], a factor analysis with eight fixed factors was performed. Despite the fact that the eigenvalues of the last three factors were smaller than 0.7, correlations between the items and their corresponding factors could be demonstrated. Only the last factor, Factor 8 (sense of control), was represented by only one item, number 4. The other items which were expected to correlate with this factor, Items 5 and 6, did not. Item 5 did not correlate strongly with any factor and Item 6 was strongly correlated with Factor 1 (intrusiveness). Interestingly, in the initial factor analysis without eight fixed factors, the two factors intrusiveness and sense of control were in the same factor. Furthermore, Item 22 correlated more strongly with Factor 4 (cognitive interference) than with the expected Factor 7 (reduced quality of life). These two factors were also summarized in one factor in the factor analysis without eight fixed factors.

Overall, the results of this study are similar to those from other studies validating the TFI in different languages [2, 10, 11]. To our knowledge, the current study is the first to validate a German version for Switzerland of the TFI. In line with the Tinnitus Research Consortium’s intention that a new international outcome questionnaire for tinnitus symptoms be developed, the German TFI for Switzerland is suggested here as a standard instrument for clinical and research settings. Despite these promising results, we have to acknowledge some limitations to our study. First, as mentioned above, we chose to randomize the items of the TFI. While we consider randomization to be an appropriate method for avoiding several sequence effects in the test construction [17], this change may have prevented us from replicating the original 8-factor structure. However, the exact impact that using a randomized TFI had on the results remains unclear and further research is needed to investigate this issue. One possibility would be to compare two groups, one having completed the original TFI and the other a randomized version of the TFI. A second limitation is that nearly 40% of patients refused to take part in the study. It is therefore not possible to confidently exclude recruiting biases. However, the distribution between the sexes in the population of those who refused to take part in the study is similar (72 female (42.4%)) to that of the participants of our study. Furthermore, the demographic and clinical data show that our population, with a mean TFI of 40.65 and a standard deviation (SD) of 23.20, is almost identical to the investigated population in the United Kingdom ([9], mean 40.6, SD 20.01). Further, compared to the populations of the original version ([8], mean 54.4, SD 24.7), the Swedish version ([2], mean 55.3, SD 19.9), and the Polish version ([25], mean 46.7, SD 22.5), the population mean and SD in our study were lower. Nevertheless, a balanced distribution of the tinnitus severity measured by the TFI categories was demonstrated in our population. The refusal rate of 40% can be partly explained with reference to the including procedure. Even before the first consultation, potential participants were asked to participate in the test and retest. This was necessary because the first consultation in our clinic includes substantial psychoeducation on tinnitus which had the potential to influence the results. Third, although the assessed test and re-test reliability were excellent, we did not investigate changes over time due to therapy. Therefore, we do not know how much change in the overall TFI score is required to be clinically significant in the German-speaking population. Further studies are needed to address this question.

## Conclusion

Taken together, our study demonstrated that the German version of the TFI for Switzerland is a suitable instrument for measuring the impact of tinnitus. The reliability and validity of this version are very good and comparable with the original version of TFI [8]. Despite the fact that in this study only five factors instead of eight could be extracted, we demonstrated relations to the original eight subscales. By this reckoning, the German version of the TFI for Switzerland can give relevant information about the different domains of tinnitus. Future studies should investigate (1) whether a non-randomized questionnaire can more accurately replicate the factor-structure of the original version, and (2) which change in the sum score of the questionnaire is required to be clinically significant in German-speaking Swiss tinnitus patients.
